# The Capricious Nature of Bacterial Pathogens: Phasevarions and Vaccine Development

**DOI:** 10.3389/fimmu.2016.00586

**Published:** 2016-12-12

**Authors:** Aimee Tan, John M. Atack, Michael P. Jennings, Kate L. Seib

**Affiliations:** ^1^Institute for Glycomics, Griffith University, Gold Coast, QLD, Australia

**Keywords:** phase variation, vaccine, DNA methyltransferase, DNA modification enzyme, gene expression, epigenetics

## Abstract

Infectious diseases are a leading cause of morbidity and mortality worldwide, and vaccines are one of the most successful and cost-effective tools for disease prevention. One of the key considerations for rational vaccine development is the selection of appropriate antigens. Antigens must induce a protective immune response, and this response should be directed to stably expressed antigens so the target microbe can always be recognized by the immune system. Antigens with variable expression, due to environmental signals or phase variation (i.e., high frequency, random switching of expression), are not ideal vaccine candidates because variable expression could lead to immune evasion. Phase variation is often mediated by the presence of highly mutagenic simple tandem DNA repeats, and genes containing such sequences can be easily identified, and their use as vaccine antigens reconsidered. Recent research has identified phase variably expressed DNA methyltransferases that act as global epigenetic regulators. These phase-variable regulons, known as phasevarions, are associated with altered virulence phenotypes and/or expression of vaccine candidates. As such, genes encoding candidate vaccine antigens that have no obvious mechanism of phase variation may be subject to indirect, epigenetic control as part of a phasevarion. Bioinformatic and experimental studies are required to elucidate the distribution and mechanism of action of these DNA methyltransferases, and most importantly, whether they mediate epigenetic regulation of potential and current vaccine candidates. This process is essential to define the stably expressed antigen target profile of bacterial pathogens and thereby facilitate efficient, rational selection of vaccine antigens.

## Introduction

Infectious diseases are a leading cause of morbidity and mortality worldwide. An estimated 23% of all deaths and 52% of deaths in children under the age of 5 years are caused by pathogenic microorganisms (1, 2). Over the past two centuries, many vaccines have been developed that aim to prime the host immune system and protect against disease. Consequently, the morbidity and mortality of many diseases have been significantly reduced, such as polio (3), or even eradicated, such as small pox (4). Vaccination is often considered one of the greatest triumphs of medical science (5).

To date, vaccines are available against 26 pathogens; with at least a further 24 vaccines in the development pipeline (6). The manufacture and composition of these vaccines varies significantly (7): from killed-whole cell or virus vaccines [e.g., Salk’s original polio vaccine (8)] and live attenuated vaccines [e.g., the measles, mumps, and rubella vaccine (9)], to “rationally designed” vaccines, which are subunit formulations specifically developed against selected cellular targets [e.g., the polysaccharide capsule-based pneumococcal conjugate vaccines (10) and the multivalent recombinant protein-based serogroup B meningococcal vaccine (11)]. The majority of available vaccines induce antibody-mediated protective immunity and target microorganisms and antigens that have little or no antigenic diversity or variability. Unfortunately, development of vaccines has been more difficult for pathogens that are antigenically diverse, as well as those that cannot be cultured in the laboratory, lack suitable animal models of infection, and/or those that are controlled by mucosal or T cell-dependent immune responses. There is an increasing need for the development of rationally designed vaccines for these pathogens, which has been facilitated by improvements in molecular biology techniques (e.g., DNA sequencing and manipulation; protein and carbohydrate purification; and chemical conjugation methods for production of multivalent vaccines) and increased understanding of pathogen biology, host–pathogen interactions, and the requirements for immunogenicity (e.g., immune correlates of protection, and the adjuvants required to elicit this protection) (12–15).

The era of “omics” and “big data” projects has unleashed a wealth of information for bacterial vaccine development, facilitating the ability to rapidly select potential vaccine antigens from genome and proteome analyses (14–17). However, antigens with variable expression, due to environmental signals or phase variation (i.e., high frequency, random switching of expression), possess inbuilt immune evasion capacity and do not make ideal vaccine candidates. Phase variation is often mediated by the presence of highly mutagenic simple tandem DNA repeats [also known as simple sequence repeats (SSRs)], and genes with these sequence features need to be identified so that can be discounted as vaccine antigens. However, recent research has identified phase variably expressed DNA methyltransferases that act as epigenetic regulators in many bacterial pathogens (18). These global epigenetic regulators, called phasevarions, can switch expression of candidate vaccine antigens that heretofore have been assumed to be stably expressed.

In this review, we provide an overview of key aspects that are important during antigen selection for pathogenic bacteria and focus on the impact of phasevarions on vaccine development.

## Key Considerations for Vaccine Antigen Selection

For rationally designed, subunit vaccines to succeed, the selection of appropriate vaccine antigens is critical. Key features of vaccine antigens include (1) immunogenicity (i.e., the ability to elicit an immune response), (2) the ability to induce protection (i.e., the ability of the elicited immune response to prevent proliferation and/or the induction of pathology by the pathogen), and (3) conservation (i.e., the presence and sequence similarity between many/all strains of the pathogen). However, the stable expression of antigens during infection is also a critical factor in antigen selection that is often overlooked.

Several “omics” approaches are now routinely used to perform systems-based screening of potential antigens, such as genome-based reverse vaccinology, proteomics, transcriptomics, glycomics, and metabolomics (14–16, 19–21). These approaches allow high throughput identification of the potential antigens of a pathogen. The subsequent analysis of antigen conservation is a relatively straightforward process and has been assisted by the increasing availability of genomes, driven by decreases in sequencing costs (22, 23). Sequence availability has also made it possible to assess antigenic drift (change by accumulation of mutations) and shift (complete replacement of antigens), both of which must be taken into account to select stable and effective vaccine antigens (24, 25).

Investigation of whether the target antigen is actually expressed by the pathogen during infection *in vivo* is a more complex task, due to regulation by environmental signals and the potential for expression to be influenced by stochastic mechanisms. The transcription and translation of cellular factors are often contingent on environmental signals (e.g., tissue tropism, pH, and temperature) and cellular conditions (e.g., cell cycle) (26–28). For example, for pathogens such as *Escherichia coli* and other enteric pathogens, entry to the site of infection induces the expression of a different antigen repertoire (29, 30) that is triggered by diverse environmental or host signals such as pH (31) and temperature (32). While methods exist that allow the identification of expressed RNA (transcriptome) or protein (proteome) content under selected conditions, data collected often only represent a single physiological state that does not always reflect conditions found in the host. Accordingly, it is important to understand when and how cellular factors are expressed, to ensure that the target antigen is expressed during infection and in the same location (i.e., during mucosal or systemic infection) as the immune response elicited by the vaccine.

## Antigen Expression and the Complication of Phase Variation

**Figure 1:**
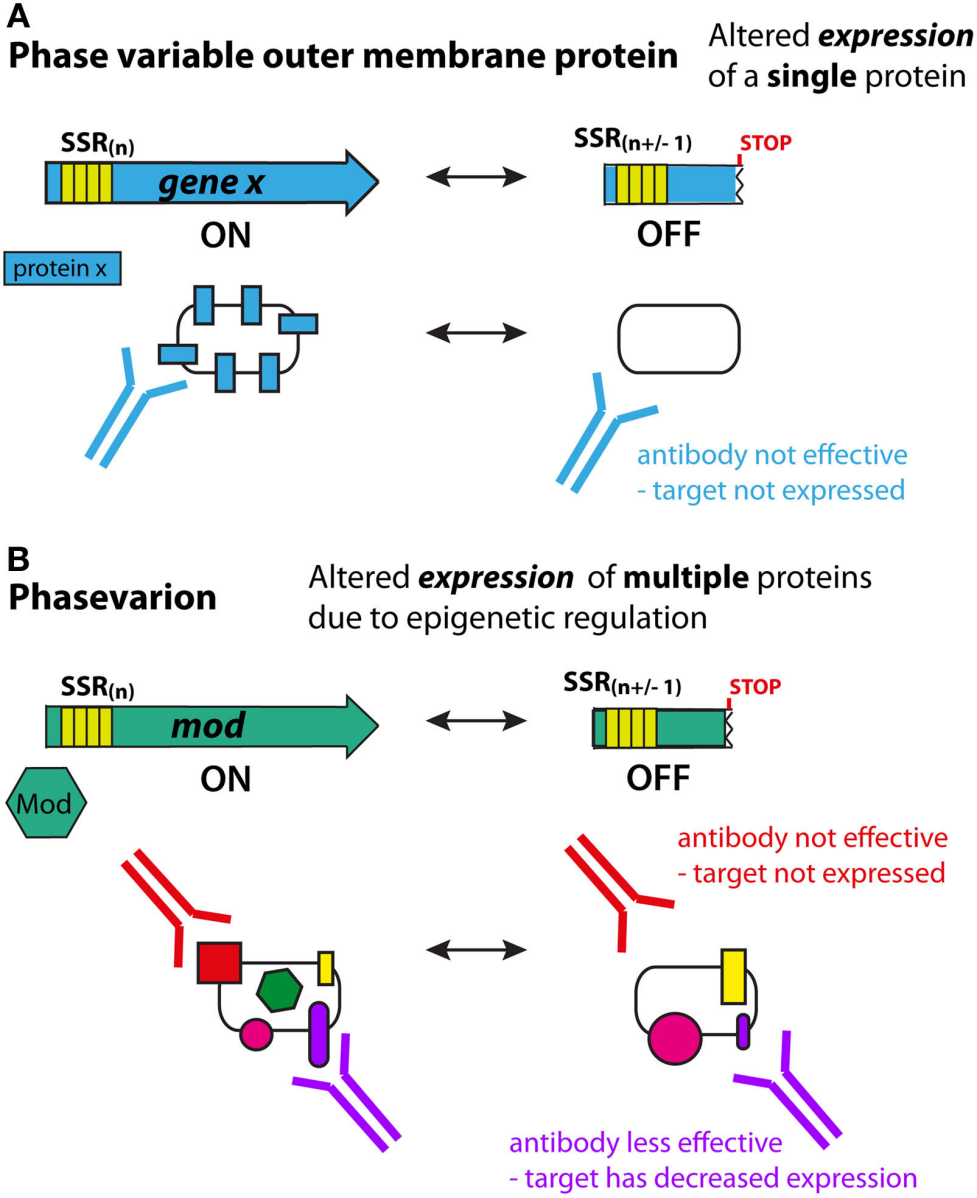
**Phase variation and immune evasion**. **(A)** For a phase-variable outer-membrane protein, slipped strand mispairing and changes in DNA sequence repeats in the gene during genome replication lead to ON/OFF expression of the encoded protein (blue). Antibodies to this antigen will not be effective if the protein has phased varied OFF. It is typically easy to predict phase-variable expression of these proteins due to the presence of DNA repeats (simple sequence repeat) in the coding region of the gene. **(B)** In phasevarions, phase-variable expression of a DNA methyltransferase causes genome-wide changes in DNA methylation, and expression differences in multiple genes due to epigenetic regulation. If these genes encode antigenic proteins/vaccine candidates, then methylation-dependent loss of expression (red protein) or reduced expression (purple protein) can lead to immune evasion as antibodies lose efficacy. However, due to the epigenetic nature of the phase-variable regulation, it is difficult to predict which proteins will have altered expression.

Phase variation is defined as the high frequency, reversible ON/OFF, or graded switching of gene expression, which is mediated through either genetic [e.g., due to variations in the number of simple tandem DNA repeats, or genome rearrangements (33, 34)] or epigenetic [e.g., *via* deoxyadenosine methylase (Dam) (35)] mechanisms at individual promoters. Many antigens in bacterial pathogens are phase variably expressed. For most phase-variable genes, switching occurs randomly during genome replication, and thus antigen expression is impossible to predict. Consequently, phase-variable components are not ideal vaccine targets since cells that have low, or no, expression of the target antigen may be able to evade the immune system (Figure 1A).

Many phase-variable genes can be identified bioinformatically, as the two main phase variation mechanisms, slipped strand mispairing and genome inversions, are well understood (36). Genes that are variable by slipped strand mispairing can be identified by the presence of multiple, tandem DNA repeats in the upstream or coding region of a gene. Slipped strand mispairing in DNA repeats causes loss or gain of repeats units, leading to frameshift mutations (ON/OFF switching) if located in the coding region, or altered expression levels if located within a promoter or operator region. In the case of genome inversions and recombination mediated mechanisms, phase-variable genes can be identified by the presence of various genetic markers such as recombinases, inverted sequence repeats, cryptic domains, and/or *via* genome comparisons for local reorganization (36, 37). Bioinformatic searches have been used successfully to identify numerous phase-variable genes in a variety of bacterial pathogens, such as *Neisseria meningitidis* (38–41), *Neisseria gonorrhoeae* (42), *Campylobacter jejuni* (43), *Helicobacter pylori* (44), and *Haemophilus influenzae* (45); and these genes are typically excluded from further screening of vaccine candidates. It is interesting to note that NadA, present in the meningococcal serogroup B vaccine (4CMenB, Bexsero), is phase variable. However, the variable expression of NadA is complex and was not easily identifiable *in silico*; the tandem repeats are distally located upstream of the *nadA* promoter and regulation involves both stochastic and classical mechanisms of gene regulation (46–48).

The DNA methyltransferase Dam is one of the best studied examples of epigenetic regulation in bacteria. While Dam itself is not phase variable or regulated, it is involved in phase variation of specific virulence genes in *E. coli* and *Salmonella*, such as *pap* (49, 50) and *agn43* (51, 52). Dam is not believed to serve as a common transcriptional regulatory mechanism (35). Rather, competition between Dam and a particular DNA-binding regulatory protein provides opportunities for competitive stochastic switches that alter gene expression at specific target sites [reviewed in Ref. (35)].

## Epigenetic Regulation of Antigens *via* Phase-Variable DNA Methyltransferases

Phase-variable DNA methyltransferases, that act as global epigenetic regulators, have been identified in a number of pathogenic bacteria and add another layer of complexity to the process of antigen selection. Phase variation of these DNA methyltransferases results in coordinated, differential methylation of the entire genome in the DNA methyltransferases ON versus OFF variants. This leads to altered expression of a set of genes that is called a phasevarion, for *phase-vari*able regul*on* (18, 53, 54) (Figure 1B). Phasevarions exert a pleiotropic effect and are associated with variable expression of proteins from diverse functional categories, such as metabolic processes, nutrient acquisition, stress responses, and virulence, as well controlling the variable expression of vaccine candidates. Phasevarions have been characterized in numerous pathogenic bacterial species, including *H. influenzae* (54–56); the pathogenic *Neisseria* (57–59); *H. pylori* (60), *C. jejuni* (43, 61), *Moraxella catarrhalis* (62, 63), and *Streptococcus pneumoniae* (64) (see Tables 1 and 2).

**Table 1:** **Phase-variable DNA methyltransferases**.

Restriction–modification type	DNA methyltransferase gene	Species	Mechanism of phase variation	Reference
I	NgoAV (*hsdS_ngoAV_*)	*Neisseria gonorrhoeae*	SSM^a^ of *hsdSNgoAV1* (poly-G repeats)	(65)
I	SpnD39III (SpnD39IIIA-FP)	*Streptococcus pneumoniae*	Recombination^b^ generates six potential *hsdS* genes (inverted repeat sequences)	(64)
I	*hsd1* and *hsd2* loci	*Mycoplasma pulmonis*	Recombination between two *hsd* loci generates eight (observed) allele combinations (*vip* and *hrs* sequences)	(66)
IIS	*cj0031*	*Campylobacter jejuni*	SSM of *cj0031* (poly-G repeats)	(43, 61)
Putative II	HpyAIV	*Helicobacter pylori*	SSM of M.Hpy.AIV (poly-A repeats)	(67)
III	*mod* (HP1407)	*H. pylori*	SSM of *res* (and downstream *mod*) (poly-C repeats)	(68)
III	*mod*	*Pasteurella haemolytica*	SSM of *mod* (CACAG repeats)	(69)
III	*modA*	*Haemophilus influenzae, Neisseria meningitidis, N. gonorrhoeae*	SSM of *modA* (AGCC or AGTC repeats)	(54–56, 58, 70)
III	*modB* (*ngoAXmod*)	*N. gonorrhoeae, N. meningitidis*	SSM of *modB* (CCCAA or GCCAA repeats)	(58, 59)
III	*modD*	*N. meningitidis, Neisseria lactamica, Neisseria mucosa, Neisseria cinerea, Neisseria polysaccharea*	SSM of *modD* (CCGAA repeats)	(57)
III	*modH* (formerly *modC*)	*H. pylori*	SSM of *modH* expression (poly-G repeats)	(60)
III	*modM*	*Moraxella catarrhalis*	SSM of *modM* expression (CAAC repeats)	(62, 63)

*^a^Slipped strand mispairing (SSM) causes frameshift mutation, altering either the DNA target specificity (type I); or the expression (ON/OFF switching) of the gene indicated. The phase-variable DNA repeat sequence is indicated in brackets*.

*^b^Genome recombination or rearrangement of domains, generating the number of alleles indicated. The sequence motifs mediating recombination are indicated in brackets*.

**Table 2:** **Phenotypes and phasevarions associated with phase-variable DNA methyltransferases**.

Allele and methylation site^a^	Species (strain)	Phenotypes^b^	Phasevarion analysis^b^	Reference
*modA1*	*Haemophilus influenzae* RdKW20	Increased resistance to heat shock	Microarray: increased expression of cysteine and glutamate/aspartate transport; heme binding; and outer-membrane protein (*opa*). Decreased expression of heat shock and chaperone proteins (*dnaJK, groEL, groES, htpG*)	(53–55)

*modA2* 5′-CCGA^Me^A-3′	*H. influenzae* 723	Increased sensitivity to ampicillin; increased biofilm formation; selection for ON *in vivo* (chinchilla infection model)	iTRAQ: decreased expression of heme utilization (HxuB, HxuC1, HemR), OMP6, and transferring binding protein 1 Microarray: increased expression of iron permeases (*hitAB, yfeACD*) and heme utilization proteins (*hxuAB*), and anaerobic respiration genes	(56)

*modA4* 5′-CG^Me^AG-3′	*H. influenzae* C486	Increased survival in opsonophagocytic killing assays	iTRAQ: OMP P2	(56)

*modA5* 5′-AC^Me^AGC-3′	*H. influenzae* 477	Increased resistance to erythromycin	iTRAQ: OMP P5	(56)

*modA10* 5′-CCT^Me^AC-3′	*H. influenzae* R2866	Increased resistance to gentamicin	iTRAQ: OMP P5, P6	(56)

*modA11* 5′-CGY^Me^AG-3′	*Neisseria meningitidis* MC58	Phenotype: increased antibiotic sensitivity	Microarray: increased expression of lactoferrin binding proteins *lbpA* and *lbpB* (potential meningococcal vaccine candidate) and other outer-membrane proteins. Reduced expression of ribosomal proteins. Altered expression of DNA repair, energy metabolism, LPS biosynthesis, and other virulence associated genes	(58, 71, 72)

*modA12* 5′-AC^Me^ACC-3′	*N. meningitidis* B6116/77	Increased antibiotic sensitivity	Microarray: increased expression of succinate dehydrogenase operon, *frpA-C* related and bacterioferritin B genes	(58, 71, 72)

*modA12*	*Neisseria gonorrhoeae* 96D551		Microarray: reduced expression of *fetA*, ferric enterobactin binding protein and putative enterobactin permease (ABC transporter)	(58)

*modA13* 5′-AGA^Me^AA-3′	*N. gonorrhoeae* FA1090	Increased association with primary cervical epithelial cells, but reduced invasion and survival. Decreased biofilm formation and antimicrobial resistance	Microarray: response to oxidative stress (*metF, metE*; *NGO0554*; *recN*), antimicrobial resistance (*mtrF*), DNA repair (*recN, NGO0318*), and amino acid biosynthesis (*metFE, NGO0340*)	(58)

*modB1* (*ngoAXmod*)	*N. gonorrhoeae* FA1090	Decreased planktonic growth, biofilm formation, and adherence and invasion of human epithelial cells	Microarray: down-regulation of biofilm-associated genes including pili (*ngo0095-98*), adhesins *mafA, mafB*, and *opaD*	(73)

*modD1* 5′-CC^Me^AGC-3′	*N. meningitidis* M0579	Increased oxidative stress resistance	Microarray: increased expression of catalase (*katA*) and factors regulated for growth in blood (*glnA, purF, proB*); decrease in cold-shock domain protein, mip-related protein homolog	(57, 71)

*modH5*	*Helicobacter pylori* P12	Not reported	Microarray: increase in *hopG* (potential vaccine candidate). Decrease in motility associated genes *flaA* and HPP12_904 (*fliK* homolog)	(60)

*modM2* 5′-GAR^Me^AC-3′	*Moraxella catarrhalis* ATCC 25239	Not reported	iTRAQ: increase in proteins important in low iron conditions (FbpA, FixC), cell adherence (RpmG, AhcY), and broth growth (LepB, NqrC); decrease in oxidative stress response (GreA, BfrA)	(62)

*SpnD39IIIA* 5′-CRA^Me^AN_8_CTG-3′	*Streptococcus pneumoniae* D39	Decreased carriage rate; selection for allele in mouse blood	RNASeq: decrease in *blp*, sucrose regulator, and fucose operon; increase in *psaABC, dnaK*	(64)

*SpnD39IIIB* 5′-CRA^Me^AN_9_TTC-3′	*S. pneumoniae* D39	Non-opaque colonies, higher phagocytosis by RAW 264.7 cells; lower blood bacteremia rates *in vivo*	RNAseq: decrease in capsule, *luxS, dexB*	(64)

*SpnD39IIIE* 5′-CRA^Me^AN_8_CTT-3′	*S. pneumoniae* D39	Lower bacteremia rates *in vivo*	Not reported	(64)

*SpnD39IIIF* 5′-C^Me^ACN_7_CTT-3′	*S. pneumoniae* D39	Lower bacteremia rates *in vivo*	Not reported	(64)

*cj0031* 5′-CCYG^Me^A-3′	*Campylobacter jejuni* NCTC11168	Enhanced adhesion and invasion of epithelial cells; increased biofilm formation; and increased phage restriction ability. ON strains are selected for *in vivo* (chicken model)	Not reported	(61)

*^a^Where available, the methylation site has been indicated by ^Me^ preceding the methylated residue (underlined)*.

*^b^Unless otherwise specified, the phenotype and phasevarion changes described are increased when the DNA methyltransferase is in phase ON versus OFF. The method used for phasevarion analysis and examples of genes regulated are given*.

Phasevarions present a critical challenge for vaccine development, in that the genes controlled by phase-variable DNA methyltransferases do not have easily identifiable markers to indicate their phase-variable expression – these markers are only associated with the DNA methyltransferase and not the genes it regulates. Consequently, these components may be considered as potential vaccine candidates because their expression is erroneously assumed to be stable. This could potentially result in less effective, or completely ineffective, vaccines (Figure 1B).

## Types of Phase-Variable DNA Methyltransferases

**Figure 2:**
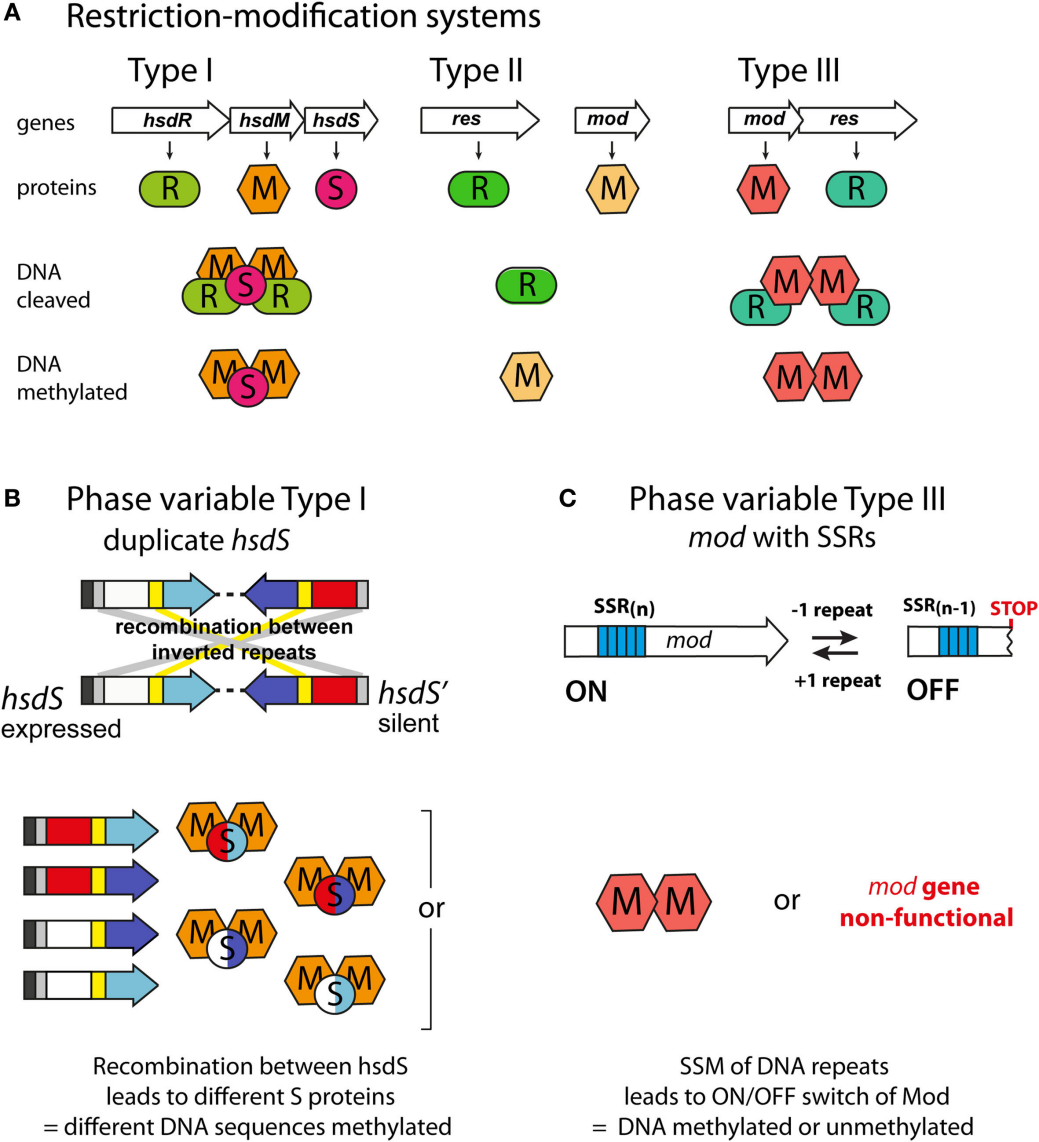
**Phase-variable DNA methyltransferases**. **(A)** The three main types of restriction–methylation (R–M) systems: type I consists of separate restriction (R), methyltransferase (M), and specificity (S) components, encoded by *hsdR, hsdM*, and *hsdS* genes, respectively. For restriction to occur, a pentameric R_2_M_2_S complex must form, but methylation can occur independently through a trimeric M_2_S complex. The HsdS subunits dictate the DNA sequences that are restricted and methylated. Type II systems are encoded by individual genes, often located separately on the chromosome. The resulting restriction (R) and methyltransferase (M) enzymes recognize and act independently upon the same DNA motif. Type III systems consist of colocalized *mod* [modification; encoding a methyltransferase, Mod (M)] and *res* [restriction; encoding a restriction enzyme, Res (R)] genes. Res proteins require Mod to restrict DNA (R_2_M_2_), but Mod enzymes are active as stand-alone methyltransferases (M_2_). **(B)** Phase variation of type I R–M systems *via* recombination between expressed (*hsdS*) and silent (*hsdS'*) specificity genes. Each *hsdS* gene contains two target recognition domains (TRDs), each contributing half to the sequence recognized by the HsdS protein. Shuffling of each TRD *via* recombination between homologous inverted repeats (gray at 5′ end, yellow in center) leads to four possible combinations, and therefore, four different methyltransferase specificities in this example. **(C)** Phase variation of type III R–M systems *via* slipped strand mispairing (SSM) of simple sequence repeats in the open reading frame of the *mod* genes. Loss or gain of a repeat unit leads to variation in the open reading frame and either expression of a functional Mod protein (Mod ON), or transcriptional termination through the presence of a premature stop codon (Mod OFF).

Phase-variable DNA methyltransferases have been described that are associated with all three major types of restriction–modification (R–M) systems (Figure 2A; Table 1). In type I R–M systems, the specificity of the DNA methyltransferase is dictated by a specificity subunit (HsdS). Phase variation is typically mediated by this locus, either by slipped strand mispairing [as with the NgoAV system of *N. gonorrhoeae* (65)] or by genome rearrangements of the *hsdS* subunit domains [as with the SpnD39III system of *S. pneumoniae* (64)]. In the SpnD39III system, genomic rearrangements result in recombination of one of two possible *hsdS* domain 1 sequences (TRD1.1 and TRD1.2) with one of three possible *hsdS* domain 2 sequences (TRD2.1, 2.2, and 2.3), which can result in the generation of six different *hsdS* alleles (SpnIIIA to SpnIIIF), producing six different HsdS specificity proteins (64) (Figure 2B). Accordingly, the SpnIII methyltransferase has six possible DNA specificities, each of which regulates expression of a distinct set of genes. While an individual cell expresses only one allele of each DNA methyltransferase at any particular time, populations of bacteria could express different mixtures of alleles.

In type II and III R–M systems, the DNA methyltransferases are independent proteins that dictate the specificity of the methylation site, and phase variation is typically mediated by slipped strand mispairing of SSRs in the coding sequence of the DNA methyltransferase (*mod*) gene (Table 1). Changes in repeat number cause frameshift mutations and switching of Mod protein expression between “ON” (expressed) or “OFF” (not expressed) states (Figure 2C). The type III Mod proteins are the most extensively studied (Table 1), and multiple allelic variants exist for each system, as determined by sequence differences in the DNA recognition domain responsible for methyltransferase specificity (18, 54, 56, 58, 60, 62, 71). For example, 21 *modA* alleles (56, 58, 70), 6 *modB* alleles (18, 58, 74), and 7 *modD* alleles (57, 74) have been identified to date. Unlike the type I systems described above, switching between alleles by genome rearrangement within a strain has not been reported and only one allele is present in a given strain. However, horizontal transfer of allele DNA recognition domains occurs and is postulated to generate novel DNA methyltransferase alleles over time (70, 75, 76).

## Phasevarions and Vaccine Development

The challenge for vaccine development is to determine whether specific antigens are members of phasevarions prior to investing time in developing them as vaccine candidates. Previous studies have identified proposed vaccine candidates in phasevarions, including *hopG* (encoding a major outer-membrane protein) in the *H. pylori* ModH5 phasevarion (60), *lbp* (encoding lactoferrin binding protein) in the *N. meningitidis* ModA11 phasevarion (58), HMW adhesins in the *H. influenzae* ModA2, ModA4, and ModA5 phasevarions (56), and capsule in the *S. pneumoniae* SpnIIID39B phasevarion (64) (Table 2). Furthermore, many phasevarions are associated with virulence, which may be of concern as virulence determinants are often targets of vaccine development. For example, the ModA11 and ModA12 phasevarions in *N. meningitidis* (72), ModA13 and M.NgoAX in *N. gonorrhoeae* (58, 73), and ModA2, ModA5, and ModA10 in *H. influenzae* (56) all affect antimicrobial susceptibility. ModA2 (*H. influenzae*), ModA13 (*N. gonorrhoeae*) (58), and *cj0031* (*C. jejuni*) (61) alter biofilm formation. *N. meningitidis* ModD1 can increase oxidative stress resistance and regulate factors important for growth and survival in blood (57). Different pneumococcal SpnIII alleles are associated with causing different phenotypes in *S. pneumoniae*, such as nasopharyngeal colonization (SpnIIIB) or bacteremia (SpnIIIA) (64) (Table 2).

Consequently, when considering the impact of phasevarions on vaccine development, it is important to know which allele(s) are present in the bacterial species, as well as the distribution of these alleles – that is, whether certain alleles predominate among the pathogenic strains that require targeting by the vaccine. Previous studies have used PCR and Sanger sequencing methods to identify and determine alleles (55–58, 60, 62); however, the increasing ease and lowered costs of full genome sequencing will enable the simple identification of phase-variable methyltransferases in broader, larger sample panels, as well as the identification of new or novel systems. For example, the availability of a large database of meningococcal genome sequences has recently been used to help survey the *mod* allele repertoire in over 1,600 isolates (74).

A bigger challenge lies in defining the proteins regulated within each phasevarion, as this must be determined experimentally. This has previously been accomplished by custom transcriptomic microarray analysis (57, 58, 60), but is being supplanted by next generation sequencing techniques [namely RNAseq, as in Ref. (64)] and proteomic analyses [e.g., iTRAQ, as in Ref. (56, 62)]. RNAseq allows the visualization of the full transcriptomic response to DNA methyltransferase phase variation, including differences in transcription of RNA genes (such as tRNAs) and non-coding RNAs (such as siRNAs and other regulatory RNAs). RNAseq will also provide valuable information about transcriptional start sites and upstream regulatory sequences for genes in the phasevarion, and possible transcription kinetics around methylation sites, enabling detailed mechanistic studies to be performed. In contrast, proteomic analyses will definitively identify the protein antigens differentially expressed by phasevarions under the conditions tested. This may differ from the transcriptomic data as RNA expression does not always correlate to protein translation, and so future studies should analyze expression data using multiple techniques in order to identify all members of each phasevarion. This will be invaluable for examining the actual changes in antigen levels and how this may affect vaccines.

The identification and analysis of genes controlled by phasevarions need to be carried out under conditions relevant to infection. This is because epigenetic regulation *via* DNA methylation is typically a multistep process, with DNA methylation affecting the action of regulatory proteins involved in transcription, rather than acting on transcriptional machinery itself [reviewed recently in Ref. (77)]. As such, conditions tested must be biologically relevant and allow these regulatory proteins to be active, in order to observe epigenetic regulation. This has been demonstrated by microarray analysis of the ModA11 phasevarion, where iron-limiting conditions were necessary to identify phasevarion members (mimicking iron limitation in the host, compared with standard laboratory culture conditions) (58). Unfortunately, the specific conditions that permit the full expression of the phasevarion can be difficult to determine, and bacteria should be grown under biologically relevant conditions, or if possible, collected directly from infection sites – such as from blood or mucosal surfaces. It is also critical that the whole, or representative, bacterial population is isolated and analyzed during phasevarion studies. This will allow the natural ON/OFF status and ratio of phase-variable DNA methyltransferases in the *in vivo* bacterial population to be understood.

## Concluding Remarks

The development of bacterial vaccines depends on the selection of appropriate antigens. Ideal vaccine antigens are conserved, immunogenic, and protective. They should also be consistently expressed at high enough levels during infection to be targeted by the immune system. Transient and arbitrary expression makes antigen targeting by the immune system difficult and could lead to immune evasion *via* escape of a subpopulation that do not express the antigen. For this reason, phase-variable antigens do not make ideal vaccine candidates.

Phase-variable regulators complicate the prediction of stably expressed antigens, as the regulated genes within a phasevarion lack overt markers that indicate potential random switching of expression. While phasevarions have been studied in a range of pathogenic bacteria, important questions remain regarding allele variability, distribution, and regulatory mechanisms. More detailed understanding of these factors will help to elucidate the full complement of phase-variable genes in human pathogens for which vaccine development has been problematic, and help facilitate robust antigen selection for rational vaccine design in the future.

## Author Contributions

All the authors contributed to drafting and revising the manuscript and approved the final manuscript.

## Conflict of Interest Statement

The authors declare that the research was conducted in the absence of any commercial or financial relationships that could be construed as a potential conflict of interest.
